# 

**Published:** 1860-07

**Authors:** 


*Apology.*—Owing to the serious illness of one of the editors, and the protracted absence from the city of the other, we have not been able to secure our usual variety. Accident has prevented the continuation of the translation of St. Claire-Deville's article on platinum. Its publication will, however, be resumed in the October number.

---

*New Medical Journal.*—We have received the first number of the *American Medical Times*, edited by Dr. Stephen Smith, with whom are associated Drs. Elisha Harris and George F. Shrady. In its general appearance it is equal to the best London weeklies, and reflects great credit upon all concerned. We are glad to find such full reports from the various New York Hospitals.

---

*The Journal de Chimie Medicale* contains an account of the discovery of a new and powerful sedative in neuralgia, just discovered by Dr. Field. The substance used is nitrate of oxyd of glycile, and is obtained by treating glycerine at a low temperature with sulphuric or nitric acid. One drop mixed with ninety-nine drops of spirits of wine constitutes the first dilution. It has been tried upon animals and patients with remarkable effect. A case of neuralgia, in an old lady, which had resisted every known remedy, was completely cured by this new agent. It has also been tried in dental neuralgia with equal success.

